# Supplementary material for: Design and execution of a verification, validation, and uncertainty quantification plan for a numerical model of left ventricular flow after LVAD implantation
Source: PLoS Comput Biol. 2022 Jun 13;18(6):e1010141. doi: 10.1371/journal.pcbi.1010141 (PMC9232142; doi:10.1371/journal.pcbi.1010141)
Supplement: S1 Data — This section provides the gradiation for each credibility factor and actions required on each item in the standard ASME V&V40. (PDF) [file pcbi.1010141.s002.pdf]

Design and execution of a Verification, Validation, and Uncertainty Quantification plan for a numerical model of left ventricular flow after LVAD implantation

Supporting Material 1

Alfonso Santiago[1,2], Constantine Butakoff[2], Beatriz Eguzkitza[1], Richard A. Gray[3], Karen May-Newman[4], Pras Pathmanathan[3], Vi Vu[4], Mariano Vázquez[1,2]

[1] Barcelona Supercomputing Center (BSC), Barcelona, Spain. [2] ELEM biotech, Barcelona, Spain. Email: mariano.vazquez@bsc.es. [3] US Food and Drug Administration (FDA), Silver Spring, USA. Email: richard.gray@fda.hhs.gov. [4] Department of Mechanical Engineering, San Diego State University (SDSU) San Diego, USA. Email: kmaynewm@mail.sdsu.edu.

S1 Ranking for risk informed credibility assessment

This section provides the gradiation for each credibility factor and actions required on each item in the standard ASME V&V40 [1].

1. **VERIFICATION:** Activities related to the correctness of the implementation of the numerical model.
   - 1.1. **Code verification:** Activities to ensure the correct implementation of the code.
     - 1.1.1. **Software quality assurance (SQA):** Activities to ensure repeatability and traceability of the code modifications. Ranked in three:
       - A. Very little or no software quality assurance (SQA) are followed.
       - B. SQA procedures are specified and documented.
       - C. SQA procedures are specified and documented. Quality metrics are tracked. Code anomalies are systematically registered and tracked.
     - 1.1.2. **Numerical code verification (NCV)**: Activities related to demonstrate the correct implementation and functioning of the numerical algorithms. Ranked in four:
       - A. No NCV was performed.
       - B. The numerical solution is compared to a benchmark solution from another code.
       - C. The numerical solution is compared to an exact analytical or manufactured solution, demonstrating an asymptotical approach with mesh size.
       - D. The order of accuracy is compared to the theoretical order of accuracy in an exact solution.
   - 1.2. **Calculation verification:** Estimate the numerical error in the quantity of interests (QoIs) due to spatial and temporal discretisation.
     - 1.2.1. **Discretisation error:** Estimation of error due to the finite points in time/space in which the problem is solved. Ranked in three:
       - A. No space/time convergence analysis is performed.
       - B. Space and time convergence analysis are performed obtaining stable behaviours.
       - C. Grid and space convergence analysis is performed, estimating the discretisation error.
     - 1.2.2. **Numerical solver error:** Errors originated from the numerical solution based on the solver parameters.
       - A. No solver parameter sensitivity was performed.
       - B. Solver parameters are based on values from a previously verified model.

C. A solver parameter sensitivity study is performed ensuring that the chosen values does have a negligible impact in the final model accuracy.

1.2.3. **User error:** refers to errors accrued by the practitioner (unchecked inputs).

A. Inputs and outputs were not verified.
B. Key inputs and outputs were verified by the practitioner.
C. Key inputs and outputs were verified by an internal peer review.
D. Key inputs and outputs were verified by reproducing simulations by an external reviewer.

2. **VALIDATION:** Process of assessing the degree to which the computational model is an appropriate representation of the reality for the context of use.

2.1. **Computational model:** Refers to the input of the numerical model.

2.1.1. **Model form:** Refers to the correctness of the conceptual and mathematical formulation of the computational model. Ranked on three:

A. Influence of the model assumptions are not explored.
B. Influence of some assumptions is explored.
C. Influence of every assumption is explored.

2.1.2. **Model inputs:** Refer to the values of parameters used.

2.1.2.1. **Quantification of sensitivities:** examines the degree to which the model's output is sensitive to the model inputs. Ranked on three:

A. A sensitivity analysis is not performed.
B. A sensitivity analysis of the expected key parameters is performed.
C. Comprehensive sensitivity analysis is performed.

2.1.2.2. **Quantification of uncertainties:** the degree to which known or assumed uncertainties in the model inputs are propagated to uncertainties in the simulation. Ranked in four:

A. Uncertainties are not quantified.
B. Uncertainties on expected key inputs are identified and quantified but not propagated to assess the effect in the QoIs.
C. Uncertainties on expected key inputs are identified, quantified and propagated to assess the effect in the QoIs.
D. Uncertainties on all inputs are identified and quantified and propagated to assess the effect of the simulation results.

2.2. **Comparator**: Is the data against which the simulation results are evaluated.

2.2.1. **Test samples:** Refers to the population and characteristics of the experimental subjects.

2.2.1.1. **Quantity of test samples:** examines the number of samples used. Ranked in three:

A. A single sample is used.
B. Multiple samples are used, but not being statistically relevant.
C. A statistically relevant number of samples are used.

2.2.1.2. **Range of characteristics of test samples:** This item examines the number of test conditions used. Ranked in four:

A. A single test condition is examined.
B. Test conditions in a nominal range are examined.
C. Extreme test conditions are examined.
D. The entire range of test conditions is examined.

2.2.1.3. **Measurements of test samples:** Evaluate the rigor with which the measurement data characterize each test sample. Ranked in three:

A. The test sample is not characterized (measured).
B. One or more key characteristic are measured.
C. All key characteristics are measured.

2.2.1.4. **Uncertainty of test samples measurements:** This factor examines the analysis of the uncertainty associated with the tools and methods used. Ranked in four:

A. Characteristics uncertainty not addressed.
B. Uncertainty analysis incorporates instrument accuracy only.
C. Uncertainty analysis incorporates instrument accuracy and statistics (repeated measurements).

2.2.2. **Test conditions:** evaluate the rigorousness in which the tests were executed.

2.2.2.1. **Quantity of test conditions:** Number of test conditions imposed and characterized. Ranked in two:

A. Single test condition.
B. Multiple test conditions.

2.2.2.2. **Range of test conditions:** evaluates the range of test conditions included in the comparator study. Ranked in four:

A. A single test condition is examined.
B. Test conditions representing a range of conditions near nominal range are examined.
C. Test conditions representing the expected extreme conditions are examined.
D. Test conditions representing the entire range of conditions is examined.

2.2.2.3. **Measurements of test conditions:** Examines the rigor with the measurement data that characterize the test conditions. Ranked in three:

A. The test conditions are not measured.
B. One or more key test conditions are measured.
C. All key test conditions are measured.

2.2.2.4. **Uncertainty of test conditions:** This component analyses the uncertainty associated with the tools and methods to characterize the test conditions. Ranked in four:

A. Test conditions were not characterized or their uncertainty analysis is not executed.
B. Uncertainty analysis of the test conditions characteristics incorporated instrument accuracy only.
C. Uncertainty analysis of the test conditions characteristics incorporate instrument accuracy and statistics (repeated measurements).

2.3. **Assessment:** of the accuracy of the simulation output.

2.3.1. **Equivalence of input parameters:** between the type and range of input parameters. Ranked in three:

A. The types of some inputs are dissimilar.
B. The types of all inputs are similar, but ranges were not equivalent.
C. The types and ranges of all inputs are similar.

2.3.2. **Output comparison:** Equivalency between the types of output from the computational model and those from the comparator leads to increased credibility.

2.3.2.1. **Quantity:** Quantity of QoIs to compare. Ranked in two:

A. A single output is compared.
B. Multiple outputs are compared.

2.3.2.2. **Equivalence of output parameters:** Referring to the types of outputs to be compared. Ranked in three:

A. Most types of outputs are dissimilar.
B. Most types of outputs are similar.
C. Most types of outputs are equivalent.

2.3.2.3. **Rigor of output comparison:** This refers to the method used to compare the QoIs from the computational model:

A. Visual comparison is performed.
B. Comparison is performed by arithmetic difference.
C. Uncertainty in the output of the computational model or the comparator was used.

2.3.2.4. **Agreement of output comparison:** qualitative or quantitative agreement between the QoIs in the computational model and the comparator:

A. The level of agreement is not satisfactory for key comparison.
B. The level of agreement is satisfactory for some key comparisons.
C. The level of agreement is satisfactory for all comparisons.

3. **APPLICABILITY:** Attains the relevance of the validation to support the use of the model for a determined Context of Use.

3.1. **Relevance of the Quantities of Interest for the Question of Interest**: this compares the QoIs from the validation activities to the QoIs for the context of use (CoU). Ranked in three:

A. The QoIs from validation are related but not identical to those for the CoU.
B. A subset of the QoIs from the validation are identical to those for the CoU.

C. The QoIs from the validation are identical to those for the CoU.

3.2. **Relevance of the validation activities to the CoU:** This factor summarizes the relative proximity of the CoU to the validation points. Ranked in four:

A. There was no overlap between the ranges of the validation points and the CoU.
B. There was partial overlap between the ranges of the validation points and the CoU.
C. The CoU encompassed some validation points.
D. The CoU encompassed all validation points.

References

[1] American Society of Mechanical Engineers. Assessing Credibility of Computational Modeling through Verification and Validation: Application to Medical Devices - V V 40 - 2018. Asme V&V 40-2018. 2018; p. 60.
