# Supplementary material for: Design and execution of a verification, validation, and uncertainty quantification plan for a numerical model of left ventricular flow after LVAD implantation
Source: PLoS Comput Biol. 2022 Jun 13;18(6):e1010141. doi: 10.1371/journal.pcbi.1010141 (PMC9232142; doi:10.1371/journal.pcbi.1010141)
Supplement: S2 Data — This section provides the rationale behind every achieved score for each credibility factor. The score is taken from S1 Data. (PDF) [file pcbi.1010141.s003.pdf]

Design and execution of a Verification, Validation, and Uncertainty Quantification plan for a numerical model of left ventricular flow after LVAD implantation

Supporting Material 2

Alfonso Santiago[1,2], Constantine Butakoff[2], Beatriz Eguzkitza[1], Richard A. Gray[3], Karen May-Newman[4], Pras Pathmanathan[3], Vi Vu[4], Mariano Vázquez[1,2]

[1] Barcelona Supercomputing Center (BSC), Barcelona, Spain. [2] ELEM biotech, Barcelona, Spain. Email: mariano.vazquez@bsc.es. [3] US Food and Drug Administration (FDA), Silver Spring, USA. Email: richard.gray@fda.hhs.gov. [4] Department of Mechanical Engineering, San Diego State University (SDSU) San Diego, USA. Email: kmaynewm@mail.sdsu.edu.

Nomenclature

CD/CI continuous integration and continuous deployment. 1
CoU context of use. 3
EF Ejection Fraction. 2
HR heart rate. 2
MMS method of manufactured solutions. 1
QoI quantity of interest. 2, 3
SA sensitivity analysis. 2
SQA software quality assurance. 1
UEABS Unified European Applications Benchmark Suite. 1
UQ uncertainty quantification. 3

S1 Rationale behind the achieved scores for each credibility factor

This section provides the rationale behind every achieved score for each credibility factor. The score is taken from the Supporting Material 1.

1. **VERIFICATION:**
   1.1. **Code verification:**
      1.1.1. **Software quality assurance (SQA).** *Maximum ranking: (C). Selected goal: (B). Achieved: (C).* The simulation engine used is an industrial code in constant evolution and therefore every issue and feature is tracked and metrics are periodically computed to ensure repeatably of the numerical results using a continuous integration and continuous deployment (CD/CI) pipeline [1]. The developers and expert users have the option to access the documentation and a software quality assurance (SQA) platform to report abnormal behaviour. The code is subject to international scrutiny by its inclusion in the Unified European Applications Benchmark Suite (UEABS) [1].
      1.1.2. **Numerical code verification (NCV).** *Maximum ranking: (D). Selected goal: (C). Achieved: (D).* Predictions are based on the correct implementation solution of the incompressible Navier-Stokes model [2, 3, 4]. Multiple cases of the method of manufactured solutions (MMS) were used for a grid convergence study and to evaluate the observed order of accuracy.
   1.2. **Calculation verification:**

[1] https://gitlab.com/bsc-alya/alya

1.2.1. **Discretisation error** *Maximum ranking: (C). Selected goal (B). Achieved: (C).* The simulation involves the computation of quantity of interests (QoIs) that might be sensible to spatial discretisation. Space and time convergence analysis were performed estimating the discretisation error for the problem-specific QoIs.

1.2.2. **Numerical solver error.** *Maximum ranking: (C). Selected goal: (B). Achieved: (B).* The main QoI, this is the velocity field, is robust to solver parameters. Therefore solver parameters are based on values form previous executions.

1.2.3. **User error.** *Maximum ranking: (D). Selected goal: (B). Achieved: (C).* The number of input physical parameters that are modified is reduced to: Ejection Fraction (EF), heart rate (HR), a_{VAD}, v_{VAD}, P_{LA}, R_P^{Ao}, C_P^{Ao}, and R_S^{Ao}, reducing the chance of a user error. Despite this, all inputs were verified by observation of the input files during an internal peer review.

2. **VALIDATION:**

2.1. **Computational model:**

2.1.1. **Model form.** *Maximum ranking: (C). Selected goal: (B). Achieved: (C).* The complex model involves multiple assumptions. Despite this, it's based on the known and proven equations and assumptions such as the incompressibility of the blood flow [5] or its Newtonian behaviour in the large vessels [6], which also allows using a Newtonian blood analog in the benchtop experiment..

2.1.2. **Model inputs:**

2.1.2.1. **Quantification of sensitivities.** *Maximum ranking: (C). Selected goal: (B). Achieved: (B).* Despite being a relatively reduced number of input variables the model is complex. A comprehensive sensitivity analysis (SA) was executed for the key input variables. The variables classified as deterministic will be associated with a rationale that justify such classification.

2.1.2.2. **Quantification of uncertainties.** *Maximum ranking: (D). Selected goal: (B). Achieved: (C).* The background experiment used for the validation is highly controlled and reproducible, therefore capable of obtaining statistical measures for the input variables and QoIs. Uncertainties on expected key inputs are identified, quantified and propagated to assess the effect in the QoIs.

2.2. **Comparator**:

2.2.1. **Test samples:**

2.2.1.1. **Quantity of test samples.** *Maximum ranking: (C). Selected goal: (A). Achieved: (A).* The silicone ventricle used in the bench experiment is CAD-designed and manufactured by casting. While the shape of the idealized geometry may be different between the experiments, reproducibility of the experiments given the same geometry is guaranteed.

2.2.1.2. **Range of characteristics of test samples.** *Maximum ranking: (D). Selected goal: (A). Achieved: (A).* A single test sample is used in the nominal range of key characteristics.

2.2.1.3. **Measurements of test samples.** *Maximum ranking: (C). Selected goal: (C). Achieved: (C).* The bench experiment is designed to be reproducible and in full control by the experimentalist. The ventricle is produced from a computer draw and therefore all the key characteristics of the sample are measured and easily reproduced

2.2.1.4. **Uncertainty of test samples measurements.** *Maximum ranking: (C). Selected goal: (A). Achieved: (B).* As a single test sample was used, uncertainty of characteristics is not quantified nor required.

2.2.2. **Test conditions:**

2.2.2.1. **Quantity of test conditions.** *Maximum ranking: (B). Selected goal: (B). Achieved: (B).* To ensure predictability of the computational model, multiple test conditions are evaluated.

2.2.2.2. **Range of test conditions.** *Maximum ranking: (D). Selected goal: (B). Achieved: (C).* The computational model was validated in extreme conditions for the pump speed.

2.2.2.3. **Measurements of test conditions.** *Maximum ranking: (C). Selected goal: (B). Achieved: (B).* The easy access for measurements of the bench experiment allows measuring multiple test conditions.

2.2.2.4. **Uncertainty of test conditions.** *Maximum ranking: (C). Selected goal: (B) Achieved: (A).* As the work is executed with retrospective bench data, the test conditions are not characterised nor their uncertainty analysed.

2.3. **Assessment:**

2.3.1. **Equivalence of input parameters.** *Maximum ranking: (C). Selected goal: (B). Achieved: (C).* As the model deals with classical fluid dynamics where experimental parameters are easy to measure and implement in computational model. Therefore all types and ranges of all inputs were similar.

2.3.2. **Output comparison:**

2.3.2.1. **Quantity.** *Maximum ranking: (B). Selected goal: (B). Achieved: (B).* Multiple QoIs extracted from the flow meters are compared.

2.3.2.2. **Equivalence of output parameters.** *Maximum ranking: (C). Selected goal: (B). Achieved: (C).* The goal of the current validation plan is to reproduce exactly the same measurements in the numerical model as in the experimental benchmark, therefore all the outputs are equivalent.

2.3.2.3. **Rigor of output comparison.** *Maximum ranking: (C). Selected goal: (B). Achieved: (C).* The outputs were compared with multiple validation metrics, therefore a rigorous comparison was executed.

2.3.2.4. **Agreement of output comparison.** *Maximum ranking: (C). Selected goal: (B). Achieved: (B).* Most of the characteristics had satisfactory agreement but some of them only partially agreed.

3. **APPLICABILITY:**

3.1. **Relevance of the QoIs for the Question of interest** *Maximum ranking: (C). Selected goal: (B). Achieved: (C).* The numerical model is designed to retrieve the same QoIs as the required for the Question of interest, and these QoIs quantified during the uncertainty quantification (UQ). Therefore the QoIs from the validation were identical to those for the context of use (CoU).

3.2. **Relevance of the validation activities to the CoU** *Maximum ranking: (D). Selected goal: (B). Achieved: (C).* The number of validation points were scarce due to limited available retrospective data.

References

[1] Bulla JM, Emerson A. Selection of a Unified European Application Benchmark Suite. Partnership for Advanced Computing in Europe (PRACE); 2019.

[2] Houzeaux G, de la Cruz R, Owen H, Vázquez M. Parallel uniform mesh multiplication applied to a Navier-Stokes solver. Computers and Fluids. 2013;80(1):142–151. doi:10.1016/j.compfluid.2012.04.017.

[3] Houzeaux G, Aubry R, Vazquez M. Extension of fractional step techniques for incompressible flows: The preconditioned Orthomin(1) for the pressure Schur complement. Computers & Fluids. 2011;44(1):297–313. doi:10.1016/j.compfluid.2011.01.017.

[4] Houzeaux G, Vázquez M, Aubry R, Cela JM. A massively parallel fractional step solver for incompressible flows. Journal of Computational Physics. 2009;228(17):6316–6332. doi:10.1016/j.jcp.2009.05.019.

[5] Wang S, Lee L, Lee J. A linear relation between the compressibility and density of blood. The Journal of the Acoustical Society of America. 2001;109(1):390–396.

[6] Mejia J, Mongrain R, Bertrand OF. Accurate prediction of wall shear stress in a stented artery: Newtonian versus non-Newtonian models. Journal of Biomechanical Engineering. 2011;.
