# Supplementary material for: Design and execution of a verification, validation, and uncertainty quantification plan for a numerical model of left ventricular flow after LVAD implantation
Source: PLoS Comput Biol. 2022 Jun 13;18(6):e1010141. doi: 10.1371/journal.pcbi.1010141 (PMC9232142; doi:10.1371/journal.pcbi.1010141)
Supplement: S3 Data — Results of the verification tests. (PDF) [file pcbi.1010141.s004.pdf]

Design and execution of a Verification, Validation, and Uncertainty Quantification plan for a numerical model of left ventricular flow after LVAD implantation

Supporting Material 3

Alfonso Santiago[1,2], Constantine Butakoff[2], Beatriz Eguzkitza[1], Richard A. Gray[3], Karen May-Newman[4], Pras Pathmanathan[3], Vi Vu[4], Mariano Vázquez[1,2]

[1] Barcelona Supercomputing Center (BSC), Barcelona, Spain. [2] ELEM biotech, Barcelona, Spain. Email: mariano.vazquez@bsc.es. [3] US Food and Drug Administration (FDA), Silver Spring, USA. Email: richard.gray@fda.hhs.gov. [4] Department of Mechanical Engineering, San Diego State University (SDSU) San Diego, USA. Email: kmaynewm@mail.sdsu.edu.

Nomenclature

BDF backward differentiation formula. 3
CD/CI continuous integration and continuous deployment. 2
CFD computational fluid dynamics. 4
FEM finite elements method. 2
GCI grid convergence index. 2
QoI quantity of interest. 2
RMSE root mean square error. 1, 3, 4
UEABS Unified European Applications Benchmark Suite. 2

S1 Verification

S1.1 Mesh convergence metrics

The root mean square error (RMSE) between the solution i and j is defined thought the L2 norm $||.||_2$ as:

$$\epsilon_{i,j} = ||v_i - v_j|| = \sqrt{(\tilde{v}_i - \tilde{v}_j)^2} \tag{1}$$

Once all the cases are computed and the errors $\epsilon_{1,2}$ and $\epsilon_{2,3}$ computed the observed order of convergence is calculated as [1]:

$$s_{i,k} = 1 \cdot sign\left(\epsilon_{i,j}/\epsilon_{i,j}\right) \tag{2}$$

$$q_{i,k}(p_{i,k}) = \ln\left(\frac{r_{j,k}^{p_{i,k}} - s}{r_{i,j}^{p_{i,k}} - s}\right) \tag{3}$$

$$p_{i,k} = \left[1/\ln(r_{j,k})\right]\left[\ln|\epsilon_{i,j}/\epsilon_{j,k}| + q_{i,k}(p_{i,k})\right] \tag{4}$$

Note that in our case $r_{i,j} = r_{j,k} = r = 2.0$, so $q_{i,k}(p_{i,k}) = 0.0$ and therefore the previous system of equations is reduced to:

$$p_{i,k} = \frac{\ln|\epsilon_{i,j}/\epsilon_{j,k}|}{\ln(r)} \tag{5}$$

For three velocity fields computed u_1, u_2, u_3, u_1 being the coarsest, for three subdivision levels, the order of the convergence of the numerical scheme is given by:

$$p = \frac{\ln(||u_1 - u_2||_2 / ||u_2 - u_3||_2)}{\ln(2.0)} \tag{6}$$

With the observed p value, the grid convergence index (GCI) can be computed as [1]:

$$GCI^{95\%}_{i,j} = 1.25 \frac{\epsilon_{i,j}}{r^p_{i,j} - 1} \tag{7}$$

This uncertainty estimate provides an interval $f \pm U^{95\%}$ within the true mathematical value f_T falls with a probability of 95%.

S1.2 Numerical code verification

Numerical code verification is executed as per Section 2 of [2], for a 2D Poiseuille and a 3D Womersley flow problem in a cylindrical tube. These problems have non-trivial analytical solutions that are used as true value. For both cases the discretisation error is monitored as the grid is systematically refined by halving as in [3]. If the ratio between mesh subdivisions is defined as $r_{i,j} = r_i / r_j$ then, for this case $r_{1,2} = r_{2,3} = r = 2.0$, a figure considerably larger than 1.3, the minimum value recommended [2]. The velocity field is the quantity of interest (QoI) to be verified as it is also the raw variable used calculated in the numerical model. The mesh convergence metrics are described in Section S1.1.

S1.2.1 Software Quality Assurance

The finite elements method (FEM) software is developed with a continuous integration and continuous deployment (CD/CI) strategy based on Git [1], which combines feature-driven development and feature branches with issue tracking. Git pipelines ensure continuous integration, running a series of software checks, builds, and regression tests when the developers modify the source code. The build evaluation includes 27 combinations of architectures (Intel, IBM), compilers (gnu, intel, pg, xl), and optimization options, running more than 200 regression tests executed with various MPI and OpenMP configurations, more than 4000 different executions. This method helps to detect bugs early in the development cycle and guarantee the correctness of the simulation results and the software's stability. On this manuscript Alya version 2.1 was used, available in the Unified European Applications Benchmark Suite (UEABS) [4]. Further description of the code and training material can be found in the following links [2] [3]. Following the "Credible practice of modeling and simulation in healthcare" [5], a data availability statement is included. The referenced section details how to gather the data to reproduce this work.

S1.2.2 2D Poiseuille

Problem description: This test involves a constant Poiseuille flow in a 2D rectangle.

Domain definition and space-time discretisation: The mesh is a rectangle $0.8 \times 4[cm]$, centered at and aligned with X axis. Three meshes with 12800, 51200 and, 204800 elements were used (ratio $r_{i,j} = 2.0$). For each mesh a time step of 0.01$[s]$, 0.005$[s]$ and, 0.0025$[s]$ is used respectively. The simulations run for $10[s]$ what means 1000, 2000 and 4000 time steps for each case. Density and viscosity are $\rho = 1.06[g/cm3]$ and $\mu = 0.035[Poise]$.

Initial and boundary conditions: The initial velocity domain is $v_i\mid_{t=0} = 0$. At the inlet Γ_{in} a parabolic constant flow profile with max velocity of 1.25cm/s is used, given by the formula:

$$v_x \mid_{\Gamma_{in}} = -7.8125 y^2 + 1.25 \tag{8}$$

[1] https://gitlab.com/bsc-alya/alya [2] https://www.bsc.es/research-and-development/software-and-apps/software-list/alya [3] https://compbiomedeu.github.io/applications/Alya/Alya.html

Table 1: RMSE and RMSE expressed as percentage of 1.25[cm/s] for the Poiseuille flow problem solution in 2D.

halving $[-]$	Elements $[-]$	Time step $[s]$	RMSE $[cm/s]$	RMSE [%]	$GCI^{95\%}$ $[cm/s]$
0	12,800	0.01	0.004555	0.36	0.00206
1	51,200	0.005	0.001361	0.11	0.000617
2	204,800	0.0025	0.000379	0.03	0.000171

The outlet is free with weakly imposed pressure equal to zero. The other two walls Γ_w have no slip condition $v_i\mid_{\Gamma_w}= 0$.

Analytical solution The analytical solution should follow Eq. (8).

Results: The calculation of the convergence was calculated at the nodes of the coarsest mesh, to avoid any interpolation of the solution. Results for each case are sumarised in Table 1. The observed order of convergence p was equal to 1.907, compatible with the theoretical order of convergence of 2 of the 2nd order backward differentiation formula (BDF) time scheme used.

S1.2.3 3D Womersley flow

Problem description : solve pulsatile Womersley flow in a 3D cylinder.

Domain definition and space-time discretisation: : The domain is has length $l = 4[cm]$ and radius $r = 0.4[cm]$. Three hexahedral meshes are used with 10179, 81432, and 651456 elements respectively. The time steps used for each case were 0.005$[s]$, 0.0025$[s]$, and 0.00125$[s]$ respectively. $10[s]$ were simulated, accounting for 2000, 4000 and 8000 time steps respectively. Density and viscosity are $\rho = 1.06[g/cm3]$ and $\mu = 0.035[Poise]$.

Initial and boundary conditions: The initial velocity domain is $v_i\mid_{t=0}= 0$. At the cylinder inlet a pressure boundary condition given by $P\mid_{\Gamma_{in}}= 30cos(2\pi t)[Ba]$. The outlet is free with weakly imposed pressure equal to zero. The other two walls Γ_w have no slip condition $v_i\mid_{\Gamma_w}= 0$.

Analytical solution: The velocity component along the axis of the cylinder is given by:

$$v = Re\left[\frac{A}{i\omega\rho}\left(1 - \frac{J_0\left(i^{3/2}\alpha r/R\right)}{J_0(i^{3/2}\alpha)}\right)\exp(i\omega t)\right] \tag{9}$$

where:

$$A = \Delta P/L \tag{10}$$

$$\alpha = R\sqrt{\omega\rho/\mu} \tag{11}$$

$$\omega = 2\pi f \tag{12}$$

For a representation of the analytical solution refer to the blue line in Fig. 1.

Results : Figure 1 shows the analytical (Eq. (9)) and the simulated solution. The maximum velocity reaches expected periodic behaviour after $3[s]$. Table 2 shows the RMSE of the velocity calculation with respect to the analytical solution in the middle slice ($x = 2[cm]$) for the finest mesh. A RMSE $\epsilon = 0.0[cm/s]$ at $r = 0.4[cm]$ is explained as a Diriclhet boundary condition $v_i = 0.0[cm/s]$ is imposed there what is exactly equal to the analytical solution. The observed order of convergence is $p_{\text{obs}} = 1.82$, compatible with the theoretical order of convergence of 2 of the 2nd order BDF time scheme used. Compared to the 2D Poiseuille, a larger difference is not unexpected given the 3D Womersley is 3D and transient.

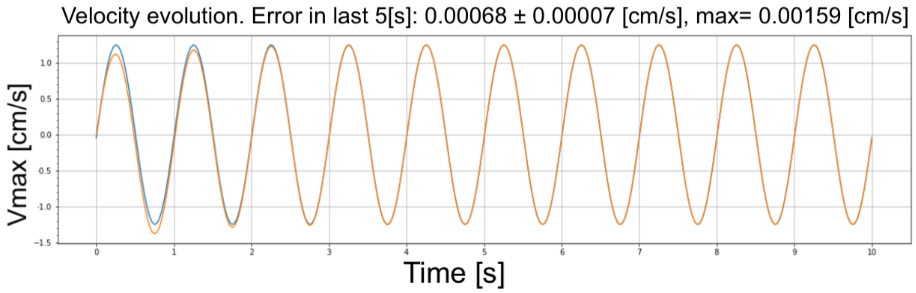

Fig. 1: Orange: Simulated maximum velocity. Blue: analytical solution.

Table 2: RMSE in $[cm/s]$ at different distances from the cylinder center at quarters of the pulsation cycle averaged radially and over the last two cycles for the finest mesh.

Time[s]	Distance from center $[cm]$				
	0.0	0.2	0.2	0.3	0.4
x.00	0.000517	0.000313	0.000236	0.000462	0.0
x.25	0.000225	0.000329	0.000311	0.000174	0.0
x.50	0.000491	0.000290	0.000253	0.000471	0.0
x.75	0.000244	0.000347	0.000324	0.000168	0.0

S1.3 Numerical calculation verification

The original model was subdivided 2 times, splitting each element into 8, to obtain meshes with $6.6M$, $53.1M$, and $425M$ elements respectively. The time step used is $\Delta t = 0.00428[s]$. If the critical time step is Δt_c then, $\Delta t/\Delta t_c = 10^{-3}[-]$.

S1.3.1 Ventricular geometry with stationary boundary conditions

The goal is to estimate the stationary error and observed convergence order for the computational fluid dynamics (CFD) solver on the mesh created for the ventricular geometry. Geometry deformation, valve model, and the pump boundary condition are turned off as they depend on measurements obtained from the CFD solver. As such geometry does not have an analytical solution, the RMSE are calculated against the finest mesh computed. Results are summarised in Table 3. The observed order of convergence is $p_{\text{obs}} = 0.971$ compatible with the theoretical order of convergence of 1 provided by the first order trapezoidal time integration.

Table 3: RMSE and RMSE expressed as percentage of the maximum speed in the domain (150$[cm/s]$) for problem specific geometry.

halving $[-]$	Elements $[-]$	RMSE $[cm/s]$	RMSE $[\%]$	$GCI^{95\%}$ $[cm/s]$
0	6.6M	1.37	0.91	1.78
1	53.1M	0.511	0.34	0.66
2	425M	0.0	0.0	0.0

S1.3.2 Ventricular geometry with transient boundary conditions

Here we show the RMSE for the complete model described in the main document.. As the complete model have transient boundary conditions, the RMSE is provided in time-averaged quantities. RMSEs are calculated against the finest mesh Results are summarised on Table 4. The time-averaged observed order of convergence is $\overline{p_{\text{obs}}} = 0.85$ compatible with the theoretical order of convergence of 1 provided by the first order trapezoidal time integration.

Table 4: Time-averaged RMSE ($\overline{\text{RMSE}}$), the timed-averaged RMSE% , and the time averaged $\overline{GCI^{95\%}}$ for the problem-specific gometry.

halving $[-]$	Elements $[-]$	$\overline{\text{RMSE}}$ $[cm/s]$	$\overline{\text{RMSE}}$ $[\%]$	$\overline{GCI^{95\%}}$ $[cm/s]$
0	6.6M	3.61	2.40	5.58
1	53.1M	2.12	1.41	3.27
2	425M	0.0	0.0	0.0

References

[1] Roache PJ. Verification and validation in computational science and engineering. vol. 895. Hermosa Albuquerque, NM; 1998.

[2] American Society of Mechanical Engineers. Standard for Verification and Validation in Computational Fluid Dynamics and Heat Transfer: ASME V&V 20. The American Society of Mechanical Engineers (ASME). 2009;doi:10.1016/S0160-4120(97)89943-6.

[3] Houzeaux G, de la Cruz R, Owen H, Vázquez M. Parallel uniform mesh multiplication applied to a Navier-Stokes solver. Computers and Fluids. 2013;80(1):142–151. doi:10.1016/j.compfluid.2012.04.017.

[4] Bulla JM, Emerson A. Selection of a Unified European Application Benchmark Suite. Partnership for Advanced Computing in Europe (PRACE); 2019.

[5] Erdemir A, Mulugeta L, Ku JP, Drach A, Horner M, Morrison TM, et al. Credible practice of modeling and simulation in healthcare: ten rules from a multidisciplinary perspective. Journal of translational medicine. 2020;18(1):1–18.
