# Supplementary material for: Design and execution of a verification, validation, and uncertainty quantification plan for a numerical model of left ventricular flow after LVAD implantation
Source: PLoS Comput Biol. 2022 Jun 13;18(6):e1010141. doi: 10.1371/journal.pcbi.1010141 (PMC9232142; doi:10.1371/journal.pcbi.1010141)
Supplement: S4 Data — Experimental data and coefficient fitting for the pump parameters. (PDF) [file pcbi.1010141.s005.pdf]

Design and execution of a Verification, Validation, and Uncertainty Quantification plan for a numerical model of left ventricular flow after LVAD implantation

Supporting Material 4

Alfonso Santiago[1,2], Constantine Butakoff[2], Beatriz Eguzkitza[1], Richard A. Gray[3], Karen May-Newman[4], Pras Pathmanathan[3], Vi Vu[4], Mariano Vázquez[1,2]

[1] Barcelona Supercomputing Center (BSC), Barcelona, Spain. [2] ELEM biotech, Barcelona, Spain. Email: mariano.vazquez@bsc.es. [3] US Food and Drug Administration (FDA), Silver Spring, USA. Email: richard.gray@fda.hhs.gov. [4] Department of Mechanical Engineering, San Diego State University (SDSU) San Diego, USA. Email: kmaynewm@mail.sdsu.edu.

S1 Calculation of the pump input variable ranges

While the physical pump has the rotor speed as input, its operation is described through the pressure-flow curve, or simply the H-Q curve. The H-Q curve is a function of the pump speed and the characteristics of the inlet and outlet tubings, as these tubing affect the system pressure drop. To fit the pump H-Q curve model (Equation 3 in main document), the San Diego State University (SDSU) recovered the H-Q performance curves of the used pump for multiple speeds. These measurements are shown as marks in Fig. 1. These measurements were fitted by a non-linear least squares method to a second order polynomial to obtain the left ventricular assist device (LVAD) pump coefficients (Table 5 in the main document). As, for this data $c_{\mathsf{VAD}} \sim 0.0$ in every case, the quadratic coefficient was forced to zero $c_{\mathsf{VAD}} \equiv 0.0$. The fittings are shown as lines in Fig. 1. The fitting error ϵ_{fit}, shown as a light gray area in Fig. 1, is calculated as the square root of the diagonal of the fitting covariance matrix, also called the standard deviation. To account for experimental error ϵ_{exp} we include a 10% error range for each coefficient, shown as dark gray in Fig. 1.

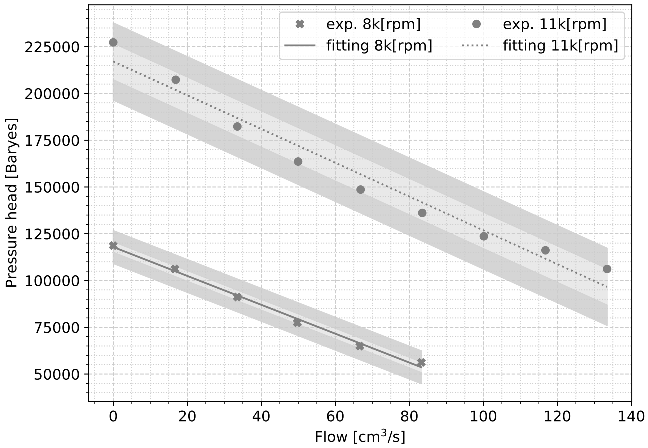

Fig. 1: Different H-Q curves measured for the pump operating at multiple speeds $(5k, 8k, 11k, 14k[rpm])$. The light grey area is representing the measured fitting (numerical) error ϵ_{fit} and the dark grey area is representing the assumed experimental error ϵ_{exp}.
