# Supplementary material for: Design and execution of a verification, validation, and uncertainty quantification plan for a numerical model of left ventricular flow after LVAD implantation
Source: PLoS Comput Biol. 2022 Jun 13;18(6):e1010141. doi: 10.1371/journal.pcbi.1010141 (PMC9232142; doi:10.1371/journal.pcbi.1010141)
Supplement: Attachment — Submitted filename: Review_PCOMPBIOL-D-21-02033.pdf [file pcbi.1010141.s006.pdf]

Review of PCOMPBIOL-D-21-02033

In this study, the authors conducted modeling and simulation of left ventricular flow with LVAD on a mock-up cardiovascular system. Their goal was to demonstrate the utility of ASME V&V40 and V&V20 standards for to design and perform risk-influence analysis, and within that context, perform verification, sensitivity analysis, and validation tasks. This is a complete and therefore, exciting use case of ASME V&V40, which will be highly informative to the practitioners of modeling and simulation and those who make decisions based on simulation results. The authors may want to expand documentation of the burden as part of this goal. This is reported (in terms of computational cost) and noted as expensive as a conclusion. Nonetheless, modeling and simulation decisions in the field may be impacted by what resources are available – not only in terms of computational power but also in regard to expertise, data availability, data acquisition burden, etc. Maybe the authors can elaborate these other aspects of burden as well. From an applicability perspective, the impact of modeling and simulation is only achieved when credibility goals and resources align.

In the remainder of this document, the authors will find comments and thoughts of this reviewer organized in a linear fashion. Some clarifications and detailing of the tasks and the interpretation of their outcomes are requested. Some recommendations on organization are also provided.

In abstract:

"small numerical errors" – Please refrain using adjectives such as "small" that may be ambiguous. You may want to report what the error is and why you consider it negligible.

In data and code availability:

The authors may want to refer to the specific version of the code that was utilized for the analysis and maybe create a package with DOI to ensure permanent access to the code. This includes both Alya and Dakota.

Data dissemination should be strengthened to increase the potential impact of the study, inline with dissemination guidance as part of credible practices of modeling and simulation[1]. Data sharing can be performed by depositing the data sets in public repositories (providing DOIs), possibly along with publishing a data descriptor.

Model dissemination should be considered as well (geometry, meshes, markup). With the dissemination of the model (along with code and data), the manuscript and related assets will be a complete training tool to develop and perform modeling and simulation and VVUQ in cardiovascular systems. This will also increase the reproducibility potential of the study. Model sharing can be performed by depositing the model in public repositories (providing DOIs), possibly along with publishing a model descriptor.

In introduction:

"model credibility": - The use of the term "model credibility" without elaborating on what the authors mean is ambiguous, i.e. analogous to saying model validity without elaborating for what and under

---

[1] https://doi.org/10.1186/s12967-020-02540-4

which conditions. Credibility is a broader term inclusive of all practices of modeling and simulation (including VVUQ but not limited to those)[2].

In 2.1 description of the benchtop model:

Please describe the physical representation of the "body" in the bench system that corresponds to the the systemic circulation parameters -> "a 3-element Windkessel model with RAop = 1.7×1 × 108 [P a · s/m3], CAop = 1.2×1 × 10−8[m3/P a], RAos = 7.8×1 × 106[P a · s/m3], following the method in [21]." Correspondence of these to the physical system will be a source of uncertainty. This may be further elaborated in section 2.2.3 initial and boundary conditions.

In 2.2.1 overall simulation pipeline:

What informed the decision for initial mesh densities? -> "The meshes have 200k for the solid and 1.6M elements for the fluid."

Please elaborate on what "special care" means? Also, it looks like a reference is missing. -> "Special care was taken with the fluid mesh, including a boundary layer valid for Re < 4000[−]"

Please elaborate on why this step size is adequate. -> "For the time discretisation, a first order trapezoidal rule with a time step of 0.00428[s] was used in every case."

Please elaborate on the suitability of unidirectional FSI. It looks like pressure updates solid domain. Does solid domains deformation update fluid boundary velocities? If so, once or iteratively? (section 2.2.2 is not clear on this) How much is lost with this assumption? It may be useful to summarize quantitative information from references [15] and [24]. Also, why can't the model further be simplified to assume rigid solid boundary? -> "To obtain a computationally inexpensive and accurate way of deforming the ventricle, a unidirectional FSI [24] approach is used to deform the LV (similarly as [15]). A pressure is imposed in the external solid domain which afterwards deform the CFD domain between the ESV and the EDV."

Please also describe the modeling of the physics of solid domain, possibly as another section.

In 2.2.2 description of the CFD solver:

Please elaborate on what details of the heart model in reference [30] are relevant -> "Further details of the heart model can be found in [30]."

In 2.3 design of the VVUQ plan:

Question of interest aims to see if a selected pump speed produce valve opening and cardiac output for a range of HR and EF covering a HF patient population. This indicates that patient specificity will focus on HR and EF. Wouldn't valve model parameters and left ventricle size inform patient-specificity? If so, do any parameter sensitivity analysis implicitly cover the effects of these as emergent boundary conditions? Please elaborate.

Context of use focuses on replication of a benchtop system for parametric explorations, i.e., for preclinical design and evaluation augmenting animal and clinical studies. Will it be fair to state that the

[2] https://doi.org/10.1186/s12967-020-02540-4

context of use aims to replace (or minimize) benchmark testing? If so, a comparison of experimentation burden against computational modeling burden should be discussed later in the manuscript. If experimentation setup and a prototype device is available, which approach will be feasible? If a prototype or experimental setup is not available, will that make modeling labor and computational burden justifiable?

More elaboration in model influence will be helpful. Outcomes of animal testing and clinical trials are noted as the primary evidence for safety and efficacy of the device. It is implied that modeling and simulation can augment animal testing and clinical trials. If so, at what level the model influence becomes higher, i.e., how much reduction in animal testing and clinical trials will be possible without impacting the quality of evidence? While this is not necessarily the goal of the study, it may be useful to have a discussion later in the manuscript to guide the readers.

Also, if the modeling and simulation is aimed to accelerate design and preliminary evaluation, its influence will be high to aid design (not for regulation). Will you anticipate the context of use for the model supporting physical prototyping and bench testing?

The decision consequence implies that model failure is related to safety and efficacy of the device. Nonetheless, the context of use is focused on preclinical evaluation of designs or delivery parameters. So, in alignment with context of use, it is arguable that the decision consequence will be low, i.e. if the model is used to evaluate different designs or design settings, a failure of model may lead to economic loss, e.g. physical prototyping, animal studies, clinical trials of the wrong device or at the wrong settings. Therefore the decision consequence can be considered low to medium.

In 2.4.1 steps of the VVUQ plan:

"For the sake of brevity, the calculation and code verification tests are omitted in this text and shown in the supporting material, Section 3. The rest of the present manuscript focuses on the SA, validation, and UQ" -> A summary is warranted in here as the main focus of the study is the demonstration of ASME V&V40 for design and execution of related tasks.

In 2.4.2 sensitivity analysis:

Were the sensitivity analyses performed on "all" model inputs? Or on select ones, e.g., boundary conditions, material properties, geometry, etc.? Please provide more detail. In addition, the starting state of the model (original parameters) needs to be referred.

In 2.4.3 uncertainty quantification:

Were the parameters noted in Table 3 (indicated by the sensitivity analysis in 2.4.2) reduced and impactful parameters? If so, please refer accordingly.

What type of sampling strategy was used to propagate input parameter space to simulations? Latin Hypercube? Monte Carlo? -> "These identified uncertainties are forward propagated through the computational model down to the output to obtain the QoIs distributuons."

"QoIs distributuons" -> "QoIs distributions"

In 3 results:

This sentence fits better to methods, section 2.4.2 -> “The SA is carried out by firstly sampling the input values using latin hypercube sampling (LHS) for all the input variables. Later, these samples are used to execute independent CFD simulations.”

In 3.1.1 results of the SA:

It looks like SA was performed on a reduced number of input parameters. (also see comment above at **In 2.4.2 sensitivity analysis**). How was the decision made to evaluate only these? What about valve model parameters, solid domain compliance, etc.? -> “The SA is intended to identify the input variables with the highest impact in the QoIs. The variable ranges used for the SA are shown in Table 3.”

This section fits better to methods, section 2.4.2.

In 3.1.3 discussion of the SA results:

The authors are encouraged to check work by Marsden and colleagues[3]. Their studies on uncertainty propagation in hemodynamics will be relevant in light of the goals of this study -> “While SA is a common tool other fields of cardiac modelling like electrophysiology or solid mechanics [39, 40, 41], there is no published work with a local nor a global SA for ventricular CFD.”

“The unexpectedly [45, 44] small influence of the mean atrial pressure (PLA) and arterial impedance (characterised via RAo P , RAo S , and C Ao P) can be explained due to the lack of Frank-Starling mechanism [46] in the silicone ventricle of the experiment and therefore also in its computational analogue” -> This brings the question of transferability of VVUQ to patient simulations. Please elaborate, particularly in view of the context of use of the model.

In 3.2.1 validation points and ranges:

An error range of 10% was assumed for EF and HR and for measurement uncertainties of QoI. While this is understandable given the lack of information, the level seems arbitrary, which is also matching between inputs and outputs. Can the authors provide an indication of the interaction between the magnitude of model input errors and measurement uncertainty?

In 3.2.4 discussion of the UQ results:

Can aortic valve model parameters (maximum possible porosity, reference pressure gradient) contribute to this? -> “If the aortic valve flow QAo is plotted for each execution (not shown in this manuscript) the clustering stands out as a set of curves where the Aortic valve fully opens, allowing flow through it.”

It will be worth describing under what conditions the pump may need to operate further from the middle operation conditions. This may relate to adverse events or certain phenotypes of patients in regard to LVAD design and patient selection for such design. -> “The lack of publications with UQ analyses for ventricular CFD makes comparing these results a difficult task. The results suggest a mismatch between the experimental and simulation results for the 0k[rpm] (LVAD off) and the 11k[rpm] cases. These validation points highlight potential issues that are worth exploring.”

In 3.3 discussion on the V&V40 credibility factors:

---

[3] https://doi.org/10.1016/j.cma.2020.113030

Please provide section numbers for sub-headings, e.g. for “Verification credibility factors.”

“The CoU does not require evaluating the QoIs for different geometries, therefore a single idealised geometry was considered for the credibility evidence.” -> If CoU is aimed for evaluating design for different patients, evaluation of different geometries will be a requirement. Please elaborate.

“achieving a C out of B” -> “C out of D”

“the goals where surpassed” -> “the goals were surpassed”

“did not obtained” -> “did not obtain”

In 4 application to ramp study

“most LVAD patients experience changes in cardiac geometry and function during the use of LVAD support.” -> Another indicator of why different geometries may need to be evaluated in sensitivity analysis or V&V.

It seems like the application to ramp study will be a future context of use of the model, beyond the model’s use for preclinical device evaluation augmenting/reducing bench testing. This will certainly change the risk determined by model influence and decision consequence. The transferability of knowledge gained in this study (in regard to VVUQ) to such a context of use warrants further discussion.

In conclusion

“While [51] reviews the use of computer model for critical health applications under the ASME V&V40 standard [10], this work presents for the first time a complete execution of a VVUQ plan following the cited guideline.” -> This is the major and possibly most impactful contribution of the manuscript in the perspective of regulatory science. Maybe highlight at the beginning of the conclusion.

In A Calculation of the pump input variable ranges

This section can be provided in a file for supporting information.

Figures & Tables:

Figure 1 missing (the referrals in text start with Figure 2)

Order of referrals to figures in text is not sequential (Figure 14 referred in section 2.1, Figures 5 and 9 in section 2.2.1, etc.)

Order of referrals to tables in text is not sequential (Table 4 referred in section 2.1, etc.)

Table 2, in the column evaluation goal, please direct the reader to the section where the activity is described in more detail and the conclusion on the grade of activity can be inferred.

Table 3, “Aortic parallel resistnace” -> “Aortic parallel resistance”

In Supplementary Material S2:

Please provide any relevant citations to support statements in 1.1.1 and 1.1.2.

Please provide any relevant citations in regard to previous executions to support statement in 1.2.2.

In 1.2.3 User error, it is arguable that ventricle geometry, solid domain properties, fluid domain properties and boundary conditions will result in more than 5 parameters. Also, how was the internal review performed?

Please provide any relevant citation to strengthen the statement, “known and proven equations”, in 2.1.1.

Based on the descriptions in the manuscripts and the comment above, the achieved ranking on 2.1.2.1 seems to be *B. A sensitivity analysis of the expected key parameters is performed.*

Based on the descriptions in the manuscript and this reviewer’s comments above, the achieved ranking on 2.1.2.2 seems to be *C. Uncertainties on expected key inputs are identified, quantified and propagated to assess the effect in the QoIs.*

In 2.2.1.1, while the silicone ventricle is CAD geometry, it is idealized therefore propagating the assumptions of what idealized geometry should be. Therefore, the statement “no more than one silicone ventricle” is not strongly justified.

In 2.3.2.4, it is arguable that achieved ranking is *B. The level of agreement is satisfactory for some key comparisons* based on the statement “Most of the characteristic had satisfactory agreement but some of them only partially agreed”. Also, “(CD)”-> “(C)”

In Supplementary Material S3:

In S3.2.1, please cite relevant publications and websites to strengthen the source of information provided in here.

In S3.3, the authors note three mesh densities for the model: 6.6M, 53.1M, and 425M elements. In the manuscript, they note “The meshes have 200k for the solid and 1.6M elements for the fluid.” Did the sensitivity analysis, and validation with UQ performed with the finest mesh density? Or, did the error rate due to mesh convergence propagated to uncertainty analysis?

Can the authors comment on any convergence analysis using the full model in regard to time discretization?
