# Supplementary material for: Design and execution of a verification, validation, and uncertainty quantification plan for a numerical model of left ventricular flow after LVAD implantation
Source: PLoS Comput Biol. 2022 Jun 13;18(6):e1010141. doi: 10.1371/journal.pcbi.1010141 (PMC9232142; doi:10.1371/journal.pcbi.1010141)
Supplement: Attachment — Submitted filename: PLOS_answer_to_reviewer2.pdf [file pcbi.1010141.s007.pdf]

Answers to Reviewer #2 comments

R2-Comment1 (R2-C1): The authors presented a very through approach for the verification, validation and uncertainty quantification of a model of the LV-LVAD system. It is a first step towards the possible use of such simulations for in silico clinical trials.
However, even tough the study is overall complete and well designed, I would like to highlight a few points that would require further clarifications.

Author's Answer - Comment1 (AA-C1): We are thankful with R2 for the time and effort spent in this thorough review.

R2-C2: General comments. Could the authors please use units that are more commonly used in clinical practice (cardiac output in L/min and pressure in mmHg)?

AA-C2: PLOS submission guidelines state that the manuscript should use the international system units. We believe a trade-off solution is to change the values to the international unit system, followed with a conversion to the clinical units (mmHg and L/min) for the section most relevant to the practitioners (results and aplication).

R2-C3: 2.1. Could the authors please comment on the adequacy between the HM2 inflow cannula and the tygon tubing they used in the experiments?

AA-C3: The original inflow cannula is used, and the tygon tubing replaces the original outflow graft of the heartmate II. Tygon tubing is used because the standard outflow graft is a porous vascular graft material - same that is used for other vascular grafts. It leaks by design but in vivo the blood micro clots seal the graft but we have to replace it with a non-leaky tube of the same diameter.

R2-C4: 2.1. The authors have retrieved the H-Q curves for each pump speed. Was it done for a "steady" state? Could the authors provide more information about the protocol they implemented to get these curves? They are the standard for LVADs but a few studies have also observed that the LVAD response is more complex and unsteady, which might explain some discrepancies between the model and the experiments.

AA-C4: Yes, it was done for steady state, for each point of the curve. The HQ curve is the transfer function that characterises the pump behaviour, a "signature" of how it performs. The standard way of retrieving the HQ curve is to measure the pump flow for increasingly larger pressure heads. LVADs with the so called "speed modulation" or "artificial pulse" have a varying speed that leads to an HQ curve that changes with time. The heartmate II is a continuum flow pump, meaning that for a predefined speed, the HQ curve will remain the same.

R2-C5: 2.2.1. Could the authors give more details about the solid mesh they used for the one-way FSI? (shell elements or solid elements?) What mechanical properties have they used for the solid model? (only the young modulus is given in the description of the experiments). Have the authors compared the overall mechanics of the solid domain between their model and the experiments?

AA-C5: We have a similar comment from Reviewer 1. The following text has been added to the document to address the reviewer's concern: "*The solid mechanics mesh is created directly from the original geometry, containing at least four linear tetrahedra in the wall thickness, obtaining a total of 200k elements*". Also "*The FSI problem can be tackled by a unidirectional or a bidirectional approach [25]. In the former approach, the solid problem unilaterally deforms the fluid mesh. In the latter approach, an iterative process is required to balance the internal forces of the solid problem with the surface pressure of the fluid problem. To obtain a computationally inexpensive and accurate way of deforming the ventricle, a unidirectional FSI approach is used to deform the LV. This same approach is used in [15], where a pressure is imposed in the external solid domain which afterwards deforms the CFD domain between the ESV and the EDV. In this unidirectional FSI approach the solid domain is exclusively used to impose the boundary deformation and velocity in the fluid domain, but no force is applied back to the solid problem, as it would be in an iterative bidirectional FSI formulation [25]. We justify this choice from the working principle of the experimental set-up (Section 2.1). The piston forces volume changes in the silicone ventricle independently of the internal ventricle pressure.*" Finally: "*Solid mechanics is modelled via linear momentum balance [31], using a neo-Hookian formulation to represent the Platinum-cured silicone [32]. As described in Section 2.1, the solid material bulk modulus is K = 1000[kPa] and its shear modulus is G = 200[kPa]. The implicit formulation is solved with a GMRES algorithm*"

R2-C6: 2.2.3. Could the authors explain their choice to keep the mitral valve inlet at a constant pressure? To my understanding, this is not the case for the experiments. Overall, a sketch of the FSI model with the different BC would really help us understand the simulation.

AA-C6: On the contrary, for the benchtop setup the mitral valve pressure remains almost constant during the experiments. The following sentence is added to address the reviewers comment: "*Constant mitral pressure is achieved by using a large open reservoir for the LA, maintaining the LA pressure constant through the studies*" Figure 1 was intended to be a description of the experimental inputs used in the simulation. Figure 1 was modified and the caption detailed further to address the reviewer's question. We include measuring points for the different QoIs in the experiment and the simulation. Arrows represent the flow direction and ventricular deformation. The caption was modified to the following, more descriptive, text: "*Leftmost side: Schematic of the experiment setup. LA: left atrium, MV: Mitral valve, AV: Aortic valve, Ao: aorta. Flow and pressure sensors are indicated in blue and red respectively. The body lumped system is afterwards characterised with a three element Windkessel model with parameters R_p, R_s and C_p. Center: variables extracted from the benchtop experiment to create the simulation and address theUQ. Rightmost side: Schematic of the simulation, including the lumped models for the boundary conditions. The measured P_LA is imposed in the mitral valve. The measured R_p, Rs and Cp are used in a three element Windkessel boundary condition at the Aortic valve output. The H-Q curve retrieved and measured is used as a dynamic boundary condition in the LVAD outflow. The benchtop piston dynamics is used as boundary condition for the deformable LV geometry.*"

R2-C7: 2.2.4. The authors used a porous layer to model the valve, which is numerically more efficient. Have they assessed the effect of this model assumption on their results? In

the experiments, the valve motion may impact the intraventricular flow patterns, especially the opening of the aortic valve.

AA-C7: We agree with the reviewer that the porous valve model can (and does) have an impact in the internal LV fluid dynamics. Before providing further insights into this topic let us state that the Context of Use (CoU) and Quantities of Interest (QoI) have been specifically designed targeting mass flow through the boundaries and not internal fluid dynamics patterns. We decided to take a mass flow comparison approach, because executing a VVUQ plan for the LV 3D CFD patterns was far beyond the current state of the art. Our latest (unpublished) model actually addresses this issue by adding an open valve geometry that is still being closed by the porous media approach. This strategy allows obtaining internal LV Eddies qualitatively similar to the experimental ones. We are currently making efforts to develop a method to compare both 3D velocity fields (experimental and simulation) that allows to qualitatively compare the results for the UQ. To summarise our answer, the valve simplification was not quantified in the QoIs because the CoU addresses mass flow through the boundaries and not LV CFD flow patterns.

R2-C8: 2.2.5. The authors have approximated the pressure-flow relationship with a quadratic equation. I assumed the coefficients AVAD, BVAD and CVAD used in equation 3 are the fitting parameters and they depend on the LVAD speed. The authors provide the values in Table 5, but it is a bit late in the manuscript. Could they please add this table to the methods section? Is this model commonly used for CFD models of LVAD?

AA-C8: We agree with the reviewer. Also In that same section there is a table showing the coefficient ranges. The table has been moved up to the methods section. Luckily, even as a medical device, LVADs behave as any other pump with their flow-pressure (H-Q) relationship being modelled as a parabola for any given time. This parabola representation does not have any non-linear or logical behviour the pump might have (e.g. sucking detection), but the pump working conditions are purposely defined to avoid the internal logic control of the pump.

R2-C9: 2.2.5. Also, have the authors evaluated the cycle do cycle dependence of their model?

AA-C9: While the answer to this question is out of the scope of the mansucript, we would like to elaborate on it. One of the reasons to use the outdated Heartmate II (HMII) instead of the newer and more popular HeartMate III (HMIII) is that the former is a continnuous flow LVAD, while the latter includes a speed modulation protocol. Speed modulation protocols (as heartmate artificial pulse or heartware´s HVAD speed modulation) periodically (once every 2 seconds) vary the pump speed in order to introduce some pulsatility and to reduce the potential stagnation regions. Unfortunately, the artificial pulse is not synchronised with the heart native pulse, producing a chaotic constructive and destructive interference in the outflow signals. The experimentalist team in our consortium already studied the effect of that speed modulation in the bench set up (https://doi.org/10.1097/MAT.0000000000001523) but the approach to reproduce that speed modulation in a computationally cheap and controlled manner in the numerical model is an ongoing discussion. If the reviewer is asking about the cycle to cycle differences with respect with the chaotic behaviour of the flow using the continnuous flow pump, the answer is simpler. The internal LV 3D CFD flows have a

chaotic behaviour. Unfortunately, this behaviour can´t be measured experimentally as the particle image velocimetry uses multiple beats to obtain a single frame of the velocity field. Therefore even if we expect a small variation of the velocity field due to the chaotic nature of the flow, it can´t be measured. In any way, 3D CFD flows are not being compared in the manuscript, but 1D time signals. The flow signals, scatter plots and cumulative distribution functions in Figures 5,6,7,9,10 and 11 capture that variation, together with the instrument error. The orange greyed areas represent the beat-to-beat measurement dispersion. Summarizing: yes the beat-to-beat dispersion in the 1D signals is being addressed in the manuscript. More complex beat-to-beat variations are left out of the scope of this manuscript due to the unsolved challenges required to measure them.

R2-C10: 2.4.2. The sensitivity analysis is an important part of this study and would benefit for further details. Could the authors comment on the relevance of the Pearson’s coefficient for their approach? Their problem is quite complex and a linear assumption, without any interactions between the variables, might be a strong hypothesis. As they state, the second approach seems more appropriate.
Also, could the authors provide the equations for the Sobol indices they compute? as they present them later in the results sections. Could they also comment on their choice of a 5th order polynomial chaos?
Finally, they stated that both analyses relied on 500 samples. How were these samples chosen (i.e. which experimental plan did the authors used? Latin hypercube sampling?)?

AA-C10: Pearson´s analysis is a popular first order approach due to its simplicity. Sobol indices calculation generally requires many more samples to calculate the high dimensionality integral or the evaluation of a reduced order model like the polynomial chaos, which is not at the reach of every research group. While Pearson´s test is simpler yet powerful and common, we think it´s useful to understand the model behaviour at a glance. The paragraph was modified to transmit that idea. We strongly believe that re-writing the integrals for the Sobol indices is out of the scope of this manuscript, not novel, and easy to reference (doi.org/10.1016 /S0378-4754(00)00270-6). Despite that, the reviewer´s comment has been addressed in the re-written paragraph. The author´s comment on the sampling method has been addressed in a comment by the reviewer 1. The paragraph was modified and expanded to the following: “*Both analyses are performed by relying on 500 samples. Pearson´s coefficient analysis is a first order approach that provides insights of the model behaviour with an accessible and straightforward method. However, it is a measure of the linear association between the inputs and the outputs and it is valid only under Pearson´s assumptions [pearson1931test] of linear and homoscedastic data with no multivariate outliers. For complex data distributions, Sobol indices are a better fitted method that provides information of the importance of each input taking into account complex factors like nonlinearities, input interactions, and sample dispersion. Sobol´s global SA rely on high-order integrals to accurately calculate the indices. These integrals require a relatively large number of samples to be evaluated. In this manuscript, the original 500 samples are used to fit a PCE emulator which is afterwards used to obtain the 5000 samples required for the Sobol integrals. The PCE polynomial order has been chosen to balance computing time and fitting accuracy. Further description on the Sobol indices and the PCE method can be found in [sobol2001global, najm2009uncertainty]*”

R2-C11: 2.4.3. The authors added a 10% error range in the QoIs measured in the experiment. Have the authors measured the repeatability and reproducibility of the experiments? If not, was this 10% error arbitrary?

AA-C11: The experiment repeatability has been widely addressed in the experimentalist laboratory publications, not only for LVADS[1], but also valves[2] and the combined devices[3]. Despite the bench experiment having been extensively tested and trusted by academic and industrial partners, a statistical analysis as required by V&V40 was not available for the data to be used in this manuscript. The reviewer may find interesting the new paragraph in the conclusion triggered by the reviewer 1, where we address the differences between bench experimentation and simulation for regulatory use and the requirements for each one.

R2-C12: Results: 3. The part of the sampling of the input values using latin hyper cube sampling belong to the methods section. (it can be repeated in the results section but needs to appear in the methods).

AA-C12: The paragraph has been moved to the methods section.

R2-C13: 3.1.1. Could the authors clarify their choice for the value ranges used for the SA? Was it based on previous studies or their experiments?

AA-C13: The SA ranges were chosen to sweep approximately one order of magnitude for the variables and to hold the validation point ranges within that of SA ranges. The sentence was modified as follows to address the reviewers comment: "*The rest of the input variables are ranged in approximately one order of magnitude, so the UQ sweeping ranges fall within the SA ranges*"

R2-C14: 3.1.1. The second approach for the SA considered nonlinear effects and interactions between the parameters. However, the authors only present the coefficient of the linear effects. If the other Sobol coefficients are not significant or very low, the authors could state it clearly in the results section.

AA-C14: Unlike the main Sobol indices, we use total Sobol indices that to account for the non linearities and the interactions between the inputs. The following sentence has been modified to ensure the reader understands the interactions are also taken into account: "*Total Sobol indices provide a more insightful tool that accounts for the effect of each input variable and their interactions in each QoI*". We agree with the reviewer that the less important inputs should also be mentioned. The following has been added to address the reviewer's comment: "*Inputs such as the Windkessel parameters R^Ao_P, C^Ao_P, R^Ao_S,*

[1] https://doi.org/10.1097/MAT.0000000000001158, https://doi.org/10.1080/0309190042000193865, https://doi.org/10.1097/01.mat.0000201961.97981.e9, https://doi.org/10.1016/j.jbiomech.2013.12.031, https://doi.org/10.1016/j.jbiomech.2015.11.049, https://doi.org/10.1097/MAT.0000000000000559, https://doi.org/10.1097/MAT.0000000000000790, https://doi.org/10.1016/j.healun.2010.06.006
[2] https://doi.org/10.1097/MAT.0b013e3181e321da, https://doi.org/10.1007/s10439-019-02218-z, https://doi.org/10.1007/s13239-016-0261-2
[3] https://doi.org/10.1016/j.healun.2010.11.007, https://doi.org/10.1097/MAT.0b013e31816a309b

and the left atrial pressure P_LA have total Sobol indices smaller than 0.25 for at least one Qoi so they are qualified as not relevant for the UQ. The reason for this is addressed in the discussion at the end of this section."

R2-C15: 3.1.2. The authors have assumed that the H-Q curves were quadratic, defined with 3 parameters, but then they only use 2 parameters in the uncertainty characterization? Was the third parameter irrelevant or very small compared to the other ones?
AA-C15: While the generic H-Q curve can be fitter with a quadratic equation, for the retrieved experimental data, the quadratic coefficient was negligible, and a straight line fits the curves better. Therefore we decided to simplify the approximation and drop the quadratic coefficient, as it can be seen in Table 5 (now Table 1).

R2-C16: Figure 4. The authors previously stated that the 4 QoIs were the max and average aortic and LVAD flows. What is QRAT that the authors present in this figure?

AA-C16: We apologize to the reviewer, QRAT is a QoI that was in the original draft but that we decided to remove afterwards as it was not providing any relevant information for the manuscript. For the reviewer's interest, QRAT is defined as QVAD/(QVAD+QAO).

R2-C17: 3.2 The authors have performed the UQ analysis for 6 experimental points. They describe them later, but could they please add a short sentence to justify this number in this section?

AA-C17: We agree with the reviewer that a clarification of this point would be useful. The following sentence was included to address the reviewer's comment: "*These six validation points are chosen to vary the QoI that provide information to answer the question of interest: EF, HR, and pump speed (via the coefficients a_VAD} and b_VAD).*"

R2-C18: Figure 6. Could the authors add units to all the plots? Also, the Qao plot is cut. Could the authors explain why they observe some flow experimentally (despite being minimal) through the LVAD, even is the RPM is 0?

AA-C18: Units were added to all plots in the manuscript, except to the Y-axis of the total Sobol indices tornado plot to ease its legibility. The "y" scale limits of the flow plots were modified to include the total QAo signal. The following paragraph is included in the UQ discussion section to address the reviewer's concern: "*On the contrary, the experiment still shows a small y-axis scattering in the LVAD QoIs (figures 6c and 10c). This is produced due to flow disturbances around the flow-meter and the sensor´s offset error (see flow-meter characteristics in Section 2.1)*"

R2-C19: 3.2.4. The authors obtained the best agreement for the 8k RPM condition, especially for the flowrate through the LVAD. However, the flowrate in the aorta is more difficult to predict, due to the complex opening and closing of the aortic valve. Could the authors comment on the capacity of their valve model to predict such phenomena correctly? Could it partly explain the difference they observed (especially in the 0K RPM condition?)?

AA-C19: We agree with the reviewer that the manuscript is missing a discussion on the effect of the valve model for the results. The sentence has been modified to address the reviewer's comment: "*The 0k[rpm] case also highlights a modelling error in the simulation results, most clearly in the flow curves in Figs. 5a and 9a: the aortic flow wave produced by the pressure curve in the model is too triangular and short-timed, and the backflow during the valve closing is too large. These differences may have been introduced by the simplified aortic valve model.*"

R2-C20: Conclusion: 5. The authors state that the bench experiments provide a highly reproducible set of comparators. Have they evaluated the reproducibility of the experiments? I assume they meant this comparatively to in vivo measurements.

AA-C20: The sentence has been modified to: "*When comparing animal experiments with benchtop experiments, the former have a larger inter-subject variability, lower reproducibility and lower access to the QoI, while the latter provides a more reproducible and accessible set of comparators.*"

R2-C21: Minor comments:

- 2.1. Please replace "during systole the ventricle contracts" by "it is contracted or compressed". This sentence may lead people to think that there is some type of active material able to contract, when the piston pump is responsible for the contraction.
- 2.4.3. Please add a space between "metric." and "To evaluate". Please remove a space before the coma in the same sentence.
- Table 3. Ejection fraction is either 0.35 or 35%.
- Figure 5. The legend of the color bar is a little difficult to read.
- 3.3. Validation credibility factors. Please add "on" after "model is based".
- Figure 6. The difference in scale in the graphs of QVAD is large and tend to be misleading as it shows large difference when they are physiologically not important. The authors could change the scale of the y axis on these plots.

AA-C21: All the comments by the reviewer were addressed except for one. While it is true that Figure 6 QVAD Y-axis is large compared to the represented signal, it is at the same scale of the QVAD plots in figures 7,8,10,11 and 12. As the referenced plots are showing the exact same quantity for the different validation points, the authors feel the urge to use the same scale for them, to ease the comparison. In this way, the final scale chosen is the one with the largest value (fig 12) and therefore the scale for some of the other plots seem large for the plotted signal. Even in that case, the plotted signal is close to zero (exactly zero in the simulation and noise in the experiment as can be seen in the scatter plots), so there is no value in reducing the Y-axis scales.

END OF THE DOCUMENT
