# Supplementary material for: Design and execution of a verification, validation, and uncertainty quantification plan for a numerical model of left ventricular flow after LVAD implantation
Source: PLoS Comput Biol. 2022 Jun 13;18(6):e1010141. doi: 10.1371/journal.pcbi.1010141 (PMC9232142; doi:10.1371/journal.pcbi.1010141)
Supplement: Attachment — Submitted filename: Review_PCOMPBIOL-D-21-02033_R1.pdf [file pcbi.1010141.s008.pdf]

Review of PCOMPBIOL-D-21-02033_R1

In this revision, the authors provided a comprehensive rebuttal to the reviewers' comments. Overall, their responses are satisfactory and complete. A few minor concerns are noted.

1. In regard to Code Availability: The authors note the possibility to provide a link to Alya source code repository. They should follow up on this and also note the version of Alya utilized in the analysis. In the rebuttal, they noted their hesitation to do this, i.e., *"For all these reasons, we do not want at all to give the reader the impression that she/he can only perform it using our code, and therefore, we do not want to (mis)lead the reader to use our code to validate anything: moreover, we want to challenge the reader to reproduce the results using third parties' codes"* The authors can have more faith in the reader and let them decide what they may want to do when empowered with such access. Particularly for the modeler in training, this may provide useful for hands on learning, at least using verification problems. The pointer to Dakota already serves for this purpose.
2. In regard to Data Availability: The authors provided geometry and mesh, which is much appreciated. These information can be utilized with model parameters noted in the manuscript to regenerate the model. Another helpful strategy maybe to provide the full description of the model, in the markup that Alya utilizes, to ensure completeness of the information in a formal language. The authors are also strongly encouraged to provide experimental data, in particular time history of pressure and flow signals (and piston velocity) as these serve as inputs or benchmarks for the system to be replicated for the CoU.
3. In regard to uncertainty levels used for experimental data: In response to Reviewer 1, the authors noted *"But assuming an arbitrary range for the input uncertinty is certinly a more accurrate "prior" than assuming a 0% experimental error. Being said this, any range would be as valid as 10%, but our choice was done based on the experimentalist criteria."* In response to Reviewer 2, they noted *"The experiment repeatability has been widely addressed in the experimentalist laboratory publications, not only for LVADS 1 , but also valves 2 and the combined devices 3 . Despite the bench experiment having been extensively tested and trusted by academic and industrial partners, a statistical analysis as required by V&V40 was not available for the data to be used in this manuscript"* The statements provided in these, including some references can be elaborated in the manuscript.
4. The authors' rebuttal includes elaborate responses to a few of comments by the Reviewer 2. Unfortunately, this Reviewer did not find similar explanations in the manuscript for (1) the motivation for the use of a porous model of the valve and future directions to address potential limitations; (2) LVAD's flow-pressure relationship; and (3) cycle to cycle dependence of the model.
