# Supplementary material for: Design and execution of a verification, validation, and uncertainty quantification plan for a numerical model of left ventricular flow after LVAD implantation
Source: PLoS Comput Biol. 2022 Jun 13;18(6):e1010141. doi: 10.1371/journal.pcbi.1010141 (PMC9232142; doi:10.1371/journal.pcbi.1010141)
Supplement: Attachment — Submitted filename: Answers_to_reviewers_it2.pdf [file pcbi.1010141.s010.pdf]

Answers to Reviewer #1 comments

R1-Comment1 (R1-C1): In regard to Code Availability: The authors note the possibility to provide a link to Alya source code repository. They should follow up on this and also note the version of Alya utilized in the analysis. In the rebuttal, they noted their hesitation to do this, i.e., "For all these reasons, we do not want at all to give the reader the impression that she/he can only perform it using our code, and therefore, we do not want to (mis)lead the reader to use our code to validate anything: moreover, we want to challenge the reader to reproduce the results using third parties' codes" The authors can have more faith in the reader and let them decide what they may want to do when empowered with such access. Particularly for the modeler in training, this may provide useful for hands on learning, at least using verification problems. The pointer to Dakota already serves for this purpose.

Author's Answer - Comment1 (AA-C1): The following was added to the data availability statement: "*The simulation software is available as part of the UEABS (https://repository.prace-ri.eu/git/UEABS/ueabs). The repository includes downloading, compiling, and test cases with details on the Alya markup.*". The Alya version has been detailed in the Software quality assurance section in the supporting material.

R1-C2: In regard to Data Availability: The authors provided geometry and mesh, which is much appreciated. These information can be utilized with model parameters noted in the manuscript to regenerate the model. Another helpful strategy maybe to provide the full description of the model, in the markup that Alya utilizes, to ensure completeness of the information in a formal language. The authors are also strongly encouraged to provide experimental data, in particular time history of pressure and flow signals (and piston velocity) as these serve as inputs or benchmarks for the system to be replicated for the CoU.

AA-C2: The following was added to the data availability statement: "*The experimental time signals for the six validation points, [...] are available at a public repository (https://doi.org/10.17605/OSF.IO/U7Z6V)*". The markup comment was addressed in AA-C1.

R1-C3: In regard to uncertainty levels used for experimental data: In response to Reviewer 1, the authors noted "But assuming an arbitrary range for the input uncertinty is certinly a more accurrate "prior" than assuming a 0% experimental error. Being said this, any range would be as valid as 10%, but our choice was done based on the experimentalist criteria." In response to Reviewer 2, they noted "The experiment repeatability has been widely addressed in the experimentalist laboratory publications, not only for LVADS 1 , but also valves 2 and the combined devices 3 . Despite the bench experiment having been extensively tested and trusted by academic and industrial partners, a statistical analysis as required by V&V40 was not available for the data to be used in this manuscript" The statements provided in these, including some references can be elaborated in the manuscript.

AA-C3: We agree with the reviewer. The following was added in the manuscript in the bench experiment description: "*The the SDSU CS has been widely used and its reproducibility addressed, not only for LVADS [7,8,9,1,10,11,12], but also valves [13, 14, 5], and with*

combined devices [2, 15]. In this work use retrospective experimental data for the validation with uncertainty quantification." Also, the following has been added in the section discussion of the UQ results to address the reviewer´s point: "*Even with a highly reproducible and tested experimental setup as the SDSU-CS (see Section 2.1) we were not able to obtain the experimental probability distributions required for the highest rankings in the ASME V&V40 standard. To be able to obtain these distributions, the exact same experiment should be repeated multiple times, a time consuming and expensive process. While working with these distributions as input data is unarguably optimal, we face the most common case where only a single experimental datapoint is available (roy2011comprehensive)*".

R1-C4: The authors' rebuttal includes elaborate responses to a few of comments by the Reviewer 2. Unfortunately, this Reviewer did not find similar explanations in the manuscript for (1) the motivation for the use of a porous model of the valve and future directions to address potential limitations; (2) LVAD's flow-pressure relationship; and (3) cycle to cycle dependence of the model.

AA-C4: The following was included to address the reviewer's comment:

1) Conclusion: "*On the simulation side, the pressure-driven porous layer is certainly affecting the LV vortical structures, as the valve geometries shapes the LV flow patterns [10,11,12]. Adding valve geometries will improve the intra-LV flow patterns in future applications where vortex quantification becomes critical*"
2) Section 2.1: "*Approximating a pump pressure-flow curve with a quadratic fit is a common engineering practice.*"
3) The following was added at the beginning of the results section: "*As the benchtop experiment uses a continuous flow LVAD, we assume there is no beat-to-beat variation. Even if there may be a change in the internal LV flow structures due to its chaotic nature, this does not affect the mass flow through the boundaries or the LV volume.*"

Answers to Reviewer #2 comments

R2-Comment1 (R2-C1): I would like to thank the authors for considering all my previous comments and modifying the manuscript accordingly. The quality of the manuscript has been improved. However, I would like to discuss a few minor points.

Author's Answer - Comment1 (AA-C1): We kindly appreciate the Reviewer´s time and effort on the reviewing process.

R2-C2: [section] 2.1. The authors used the HQ curves to model the LVAD. These curves, that the authors are also obtained experimentally, are the gold standard for LVADs. However, despite being steady pumps (with no added pulsatility events like the Heartmate 3), previous work by Noor et al. (https://doi.org/10.1111/aor.12593) have showed that under unsteady pre-load and after-load (as it is the case in this experiment) the performance of the pump (a heartmate II in the publication) is different from the HG curves obtained for steady conditions. Looking at the results that the authors presented, it is clear that the numerical simulation does not provide LVAD flowrates that are in close agreement with the

experimental flowrates. I believe that one reason for such discrepancies is the parabolic model they use for the head/flowrate relationship, which does not represent the complex unsteady performance of this steady pump subjected to unsteady pre-loads.
I am aware that these HQ curves have been used in many other CFD models for LVAD and that there is, for now, no better model, but the authors could acknowledge this as a limitation of their model. This could be part of the explanations of the discrepancies between their model and the experimental measurements.
AA-C2: The following was added to address the author´s concern: "*Using a lumped H-Q curve representation for the LVAD behaviour may also have an impact on the results. While a quadratic approximation of the pressure-flow relation in a pump is a common engineering practice, it is still a simplification that may fall apart in complex use conditions.*"

R2-C3: 2.4.2. The authors have clarified their approach for the sensitivity analysis. However, it is still unclear to me why they used Pearson's correlation, as they have not used it as a first step to isolate the most influencing parameters before implementing a more complex analysis. They could remove this analysis from the manuscript and focus on the most important part of the SA which is the determination of the Sobol coefficients. Similarly, they could move the plots of Fig.3 in the supplementary material.

AA-C3: We understand that having both metrics may seem redundant. But Pearson coefficient is simpler to understand, to implement and more intuitive. It also eases reproduction and comparison of the results. We added the following sentence to state it´s advantageous for the reader that is reproducing the results or comparing a similar work: "*While having both, Pearson coefficient and Sobol indices may seem redundant, the former is simpler to understand and implement than the latter. This eases the task of reproducing and comparing the manuscript results.*" We strongly believe that Figure 3 with the scatter plot, Pearson coefficient fittings, and Sobol indices tornado plot is one of the key results of the manuscript as it provides a quantitative representation of the system behaviour. With this the authors are doubtful of moving the figure to the supplementary material.

R2-C4: Minor comments:

1. In the introduction, the authors state that Mortality and HF status post-LVAD are associated with inefficient unloading of the LV and persistence of RV dysfunction. The authors could add here that strokes are a major problem for these patients (https://pubmed.ncbi.nlm.nih.gov/28777392/). Even if it is not the purpose of the present study, CFD could represent a great tool for the prediction of the risk of strokes.

2.1. Could the authors please remove the word "hemodynamically" in the sentence "the aortic valve remains hemodynamically closed"

2.1. Could the authors please replace Flows by flowrates, as it is the quantity that is measured in the experiments.

2.2.3 Could the authors please add "of" in the sentence "the velocity the domain deformation in imposed".

5. The sentence "This manuscript provides a thorough detail and execution of a VVUQ" is unclear. Have the authors meant: "This manuscript proved a through and detailed execution of a VVUQ"?

AA-C4:

1. The sentence was modified as follows: "*Mortality and HF status following LVAD implantation are primarily associated with inefficient unloading of the left ventricle, persistence of right ventricular dysfunction (topilsky2011echocardiographic), and stroke (adesiyun2017long).*"

2.1. the word "hemodinamically" was removed.

2.1. Every coincidence of the word "Flow" was replaced by flowrate.

2.2.3. The word "of" was added.

5. No, we want to say that the manuscript shows a detailed and complete execution of a VVUQ pipeline.

END OF THE DOCUMENT
