# Supplementary material for: Design and execution of a verification, validation, and uncertainty quantification plan for a numerical model of left ventricular flow after LVAD implantation
Source: PLoS Comput Biol. 2022 Jun 13;18(6):e1010141. doi: 10.1371/journal.pcbi.1010141 (PMC9232142; doi:10.1371/journal.pcbi.1010141)
Supplement: Attachment — Submitted filename: Review_PLOS.docx [file pcbi.1010141.s009.docx]

I would like to thank the authors for considering all my previous comments and modifying the manuscript accordingly. The quality of the manuscript has been improved. However, I would like to discuss a few minor points.

2.1. The authors used the HQ curves to model the LVAD. These curves, that the authors are also obtained experimentally, are the gold standard for LVADs. However, despite being steady pumps (with no added pulsatility events like the Heartmate 3), previous work by Noor et al. ([**https://doi.org/10.1111/aor.12593**](https://doi.org/10.1111/aor.12593)) have showed that under unsteady pre-load and after-load (as it is the case in this experiment) the performance of the pump (a heartmate II in the publication) is different from the HG curves obtained for steady conditions. Looking at the results that the authors presented, it is clear that the numerical simulation does not provide LVAD flowrates that are in close agreement with the experimental flowrates. I believe that one reason for such discrepancies is the parabolic model they use for the head/flowrate relationship, which does not represent the complex unsteady performance of this steady pump subjected to unsteady pre-loads.

I am aware that these HQ curves have been used in many other CFD models for LVAD and that there is, for now, no better model, but the authors could acknowledge this as a limitation of their model. This could be part of the explanations of the discrepancies between their model and the experimental measurements.

2.4.2. The authors have clarified their approach for the sensitivity analysis. However, it is still unclear to me why they used Pearson’s correlation, as they have not used it as a first step to isolate the most influencing parameters before implementing a more complex analysis. They could remove this analysis from the manuscript and focus on the most important part of the SA which is the determination of the Sobol coefficients. Similarly, they could move the plots of Fig.3 in the supplementary material.

Minor comments:

1. In the introduction, the authors state that Mortality and HF status post-LVAD are associated with inefficient unloading of the LV and persistence of RV dysfunction. The authors could add here that strokes are a major problem for these patients (<https://pubmed.ncbi.nlm.nih.gov/28777392/>). Even if it is not the purpose of the present study, CFD could represent a great tool for the prediction of the risk of strokes.

2.1. Could the authors please remove the word “hemodynamically” in the sentence “the aortic valve remains hemodynamically closed”

2.1. Could the authors please replace Flows by flowrates, as it is the quantity that is measured in the experiments.

2.2.3 Could the authors please add “of” in the sentence “the velocity the domain deformation in imposed”.

5. The sentence “This manuscript provides a thorough detail and execution of a VVUQ” is unclear. Have the authors meant: “This manuscript proved a through and detailed execution of a VVUQ”?
